# Abscopal effect observed in visceral and osseous metastases after liver SBRT in combination with nivolumab and relatlimab for sinonasal mucosal melanoma—a case report

**DOI:** 10.3389/fonc.2023.1143335

**Published:** 2023-04-27

**Authors:** Danielle Cerbon, Karen Moya-Brown, Ivaylo B. Mihaylov, Benjamin Spieler

**Affiliations:** Department of Radiation Oncology, Miller School of Medicine, University of Miami, Miami, FL, United States

**Keywords:** radiotherapy, abscopal, liver, effect, SBRT, immunotherapy

## Abstract

**Background:**

Primary sinonasal mucosal melanoma (SNMM) is a rare, aggressive histology usually diagnosed at advanced stages and associated with poor prognosis. Evidence regarding etiology, diagnosis, and treatment mainly derives from case reports, retrospective series, and national databases. In the treatment of metastatic melanoma, anti-CTLA-4 and anti-PD-1 checkpoint blockade increased 5-year overall survival from ~10% (prior to 2011) to ~50% (between 2011 and 2016). In March of 2022, the FDA approved the use of relatlimab, a novel anti-LAG3 immune checkpoint inhibitor, for the treatment of melanoma.

**Case presentation:**

A 67-year-old woman with locally advanced SNMM underwent debulking surgery, adjuvant RT, and first-line immunotherapy (ImT) with nivolumab but developed local progression. The patient started a second course of ImT with nivolumab and ipilimumab, but this was discontinued after two cycles due to an immune-related adverse event (irAE, hepatitis with elevated liver enzymes). Interval imaging identified visceral and osseous metastases including multiple lesions in the liver and in the lumbar spine. She went on to receive a third course of ImT with nivolumab and the novel agent relatlimab with concurrent stereotactic body radiation therapy (SBRT) to the largest liver tumor only, delivered in five 10-Gy fractions using MRI guidance. A PET/CT performed 3 months after SBRT demonstrated complete metabolic response (CMR) of all disease sites including non-irradiated liver lesions and spinal metastatic sites. After two cycles of the third course of ImT, the patient developed severe immune-related keratoconjunctivitis and ImT was discontinued.

**Conclusion:**

This case report describes the first complete abscopal response (AR) in an SNMM histology and the first report of AR following liver SBRT with the use of relatlimab/nivolumab combination ImT for metastatic melanoma in the setting of both visceral and osseous lesions. This report suggests that the combination of SBRT with ImT potentiates the adaptive immune response and is a viable path for immune-mediated tumor rejection. The mechanisms behind this response are hypothesis-generating and remain an area of active research with exceedingly promising potential.

## Introduction

Approximately 100,000 new melanoma cases (all types) were diagnosed in 2022 with nearly 8,000 estimated deaths based on American Cancer Society Statistics (1). Melanoma incidence is highest among white patients, increases with age, shows a slight female predominance, and has the largest racial survival gap among all invasive tumors, with 22% lower absolute survival for black patients (1).

Primary sinonasal mucosal melanoma (SNMM) is a rare and highly aggressive histologic subtype associated with poor prognosis. The 5-year overall survival (OS) rate is less than 25% with high post-treatment recurrence (50%–70%) (2). Evidence regarding etiology, diagnosis, and treatment outcomes is mainly derived from case reports, national cancer databases, and retrospective series (3).

Prior to 2011, chemotherapy was the mainstay of medical management for melanoma. Adjuvant treatment was limited to interferon-α2b for locoregional disease (4) and dacarbazine or high-dose interleukin-2 (IL-2) for metastatic disease (5). Interferon-α2b is associated with serious side effects (6); cytotoxic chemotherapy proved to have poor response rates, limited duration of response, and no survival benefit (7–9); and IL-2 improved OS in a small fraction of patients but with high rates of severe multiorgan toxicity (10, 11). At present, targeted agents and immunotherapy (ImT) have supplanted interferon, chemotherapy, and high-dose IL-2 as systemic treatments of choice in the adjuvant, unresectable, and metastatic settings (12, 13).

ImT has revolutionized the treatment paradigm for melanoma and many other cancers by improving treatment response and OS (1, 14–18). In metastatic melanoma, dual ImT with anti-programmed cell death protein 1 (PD-1) and anti-cytotoxic T-lymphocyte-associated protein 4 (CTLA-4) checkpoint inhibition has increased 5-year OS from 10% before 2011 to ~50% for patients diagnosed between 2011 and 2016 (19). In 2022, the United Stated (US) Food and Drug Administration (FDA) approved the use of the novel anti-lymphocyte activation gene 3 (LAG-3) agent relatlimab following results of a phase 1/2 trial and the phase 2/3 RELATIVITY-047 trial (20).

While melanoma patients show superior response to ImT regimens, treatment resistance remains common due to immune evasion, an emerging hallmark of cancer (21). There is ample preclinical and growing clinical evidence that radiotherapy (RT) as an adjunct to ImT can potentiate systemic disease response. This synergy is mainly due to RT’s ability to cause an immunogenic form of cell death that counteracts tumor immune escape mechanisms (22), and to the increasingly recognized phenomenon known as “abscopal response” (AR). The term “abscopal” (“ab”—away from, “scopus”—target) was coined in 1953 by R.H. Mole to refer to effects of ionizing radiation “at a distance from the irradiated volume but within the same organism” (23). In 2004, it was postulated for the first time that the immune system might be responsible for these “off-target” anti-tumor effects and subsequent preclinical work confirmed that AR is mediated by immunocytes (T cells). It was therefore theorized that combining immunotherapy and local radiotherapy (ImRT) could augment AR (24–26). Early case reports in melanoma trailblazed the possibility of such effects (27, 28).

Herein, we describe the case of a 67-year-old woman who presented with advanced primary SNMM, underwent debulking surgery, adjuvant RT, and an initial course of ImT, but progressed locally 2 months after RT. During her second course of ImT, she developed visceral and skeletal metastases. A third ImT course was initiated with dual anti-LAG-3 and anti-PD-1 with concurrent liver-directed stereotactic body radiation therapy (SBRT) targeting only the largest among several liver metastases. Two months after SBRT, positron emission tomography (PET)/computed tomography (CT) revealed complete metabolic response (CMR) of all intrahepatic and extrahepatic fluorodeoxyglucose (FDG)-avid disease, two cycles into the third ImT course.

## Case description

**Figure 1:**
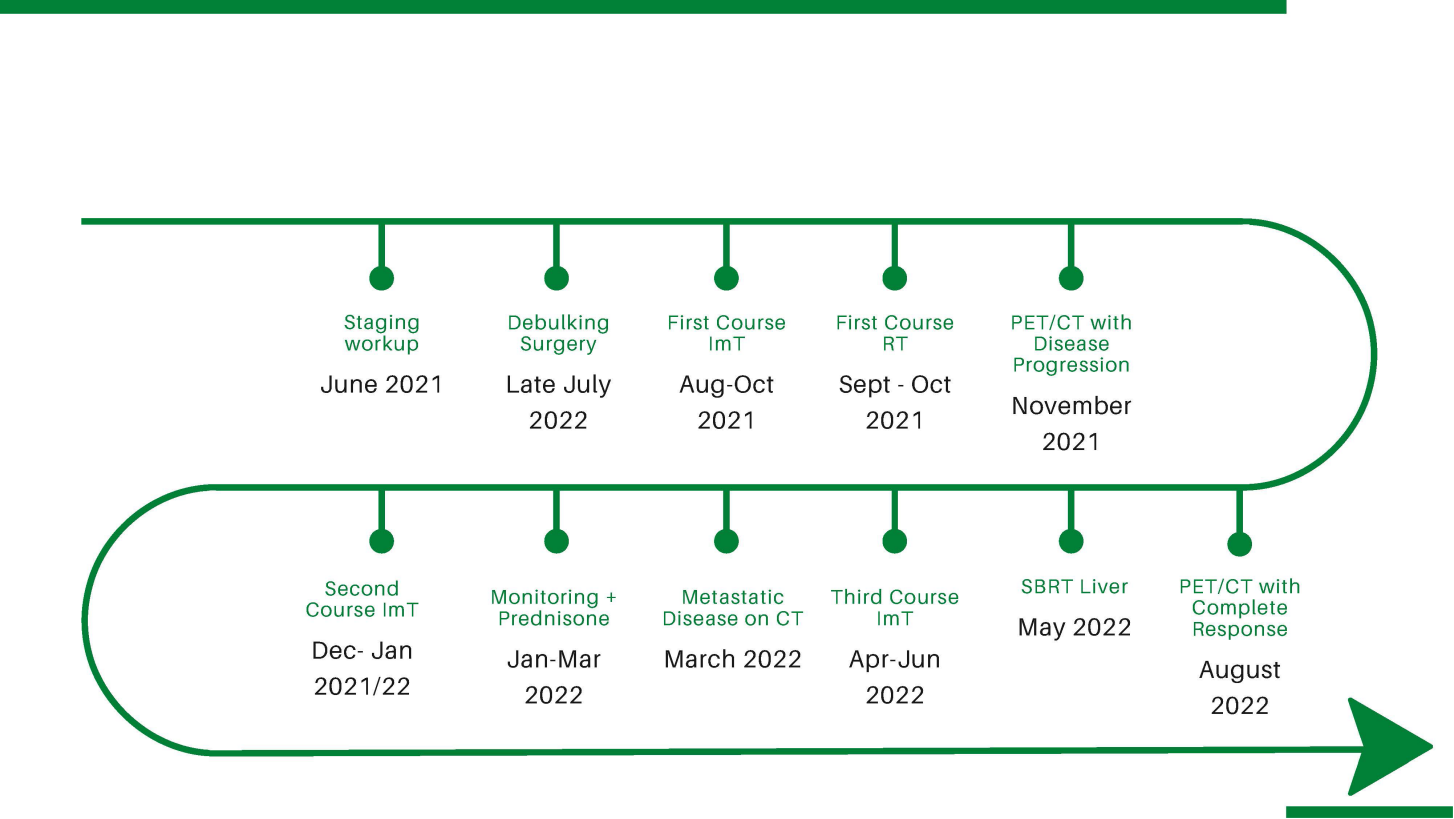
Timeline of disease development and treatment courses.

A 67-year-old woman with a history of rhinitis and coblation of the nasal turbinates presented to the emergency department for recurrent epistaxis and pain after a traumatic COVID-19 nasal swab test (see **Figure 1** for timeline).

On fiberoptic nasopharyngoscopy, a dark polypoid lesion was visualized between the middle and inferior left turbinates. Biopsy was performed and final pathology reported a heavily pigmented malignant melanoma involving the nasal mucosa, positive for S100 protein. Maxillofacial CT scan without contrast showed an abnormal soft tissue density in the left nasal cavity extending into the proximal upper left maxillary sinus and obstructing the left ostiomeatal complex. On metastatic workup, PET/CT revealed an FDG-avid right middle lobe lung nodule. The patient underwent bronchoscopy with fine needle aspiration (FNA) of the nodule. Pathology reported typical carcinoid, a finding managed separately and irrelevant to this discussion. After negative metastatic workup, the patient underwent mapping biopsies and nasal/sinus endoscopic tumor debulking. During resection, the tumor was found to extend along the nasal floor from the inferior meatus to the level of the inferior nasal septum, with pigmented mucosal changes involving the lateral aspect of the nasal vestibule, the superior aspect of the nasal septum extending onto the skull base, and the area of attachment of the middle turbinate. Pathology of the fragmented resection specimen confirmed SNMM. PD-L1 immunohistochemistry returned as high expression, intensity 3+, and tumor proportion score (TPS) ≥ 50%. The consensus recommendation at the multidisciplinary tumor board was to initiate ImT (nivolumab) and RT, to be followed by ImT (nivolumab) alone. The patient received nivolumab monotherapy (480 mg on a q4 week schedule) with concurrent intensity modulated (IM) RT to a total dose of 60 Gy in 20 fractions targeting the left nasal cavity. The patient tolerated ImT/RT well and went on to receive three cycles of adjuvant nivolumab as scheduled. However, nasal endoscopy and interval MRI identified local disease progression. Anti-CTLA-4 (Ipilimumab) was added to the ImT regimen with a plan for four cycles. After Cycle 1, the patient developed elevated liver enzymes (AST 289 U/L, ALT 301 U/L, and alkaline phosphatase 156 U/L), attributed to ImT-induced autoimmune hepatitis. ImT was discontinued and the patient was treated with intravenous solumedrol 150 mg followed by an extended course of prednisone (total duration, 30 days). Interval PET/CT showed no evidence of metastatic disease and the patient was followed closely in the clinic with radiographic surveillance. A repeat maxillofacial CT showed an increase in the size of the nodular enhancing soft tissue mass in the left posterior nasal cavity, and CT abdomen with contrast revealed a new 3.3-cm hypoenhancing liver lesion in segment 5, consistent with metastasis. CT biopsy of the liver lesion confirmed metastatic malignant melanoma. PET/CT showed rapid progression of numerous metastatic lesions within liver segments 2, 5, and 8, with the segment 5 lesion growing from 3 to 10 cm over 2 months. PET/CT also identified new FDG-avid lesions in thoracic, lumbar, and sacral vertebrae (T9, T12, L3, and S1) highly suspicious for spinal metastases.

**Figure 2:**
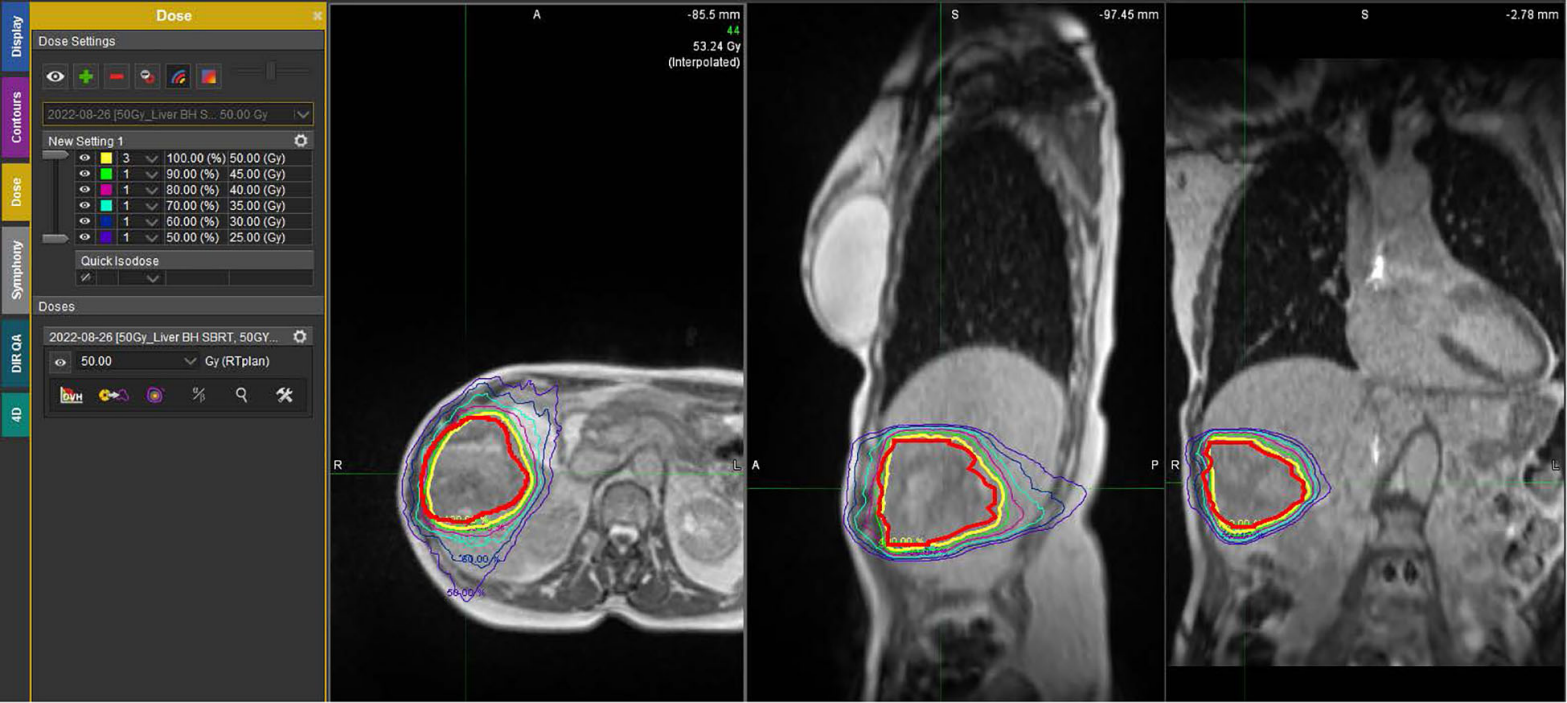
Example of the dose distribution on MRI from the SBRT course, composed of five 10-Gy fractions delivered over consecutive days. The transverse, sagittal, and coronal views demonstrate the isodose levels from 50 Gy (prescription) to 25 Gy.

After progression through two courses of ImT, the patient was presented again at tumor board and recommended to restart ImT with *Opdualag* (dual anti-PD-1 nivolumab combined with anti-LAG-3 relatlimab), and was referred to radiation oncology for concurrent stereotactic ablative radiotherapy (SBRT) of the dominant hepatic lesion. The patient received the first cycle of *Opdualag* with a concomitant course of SBRT, delivering 50 Gy in five fractions to the segment 5 liver tumor. SBRT was performed on a hybrid magnetic resonance/linear accelerator (MR/Linac) platform using MR-guided online adaptive radiotherapy (MRgOART) (29) (**Figure 2** **).**

**Figure 3:**
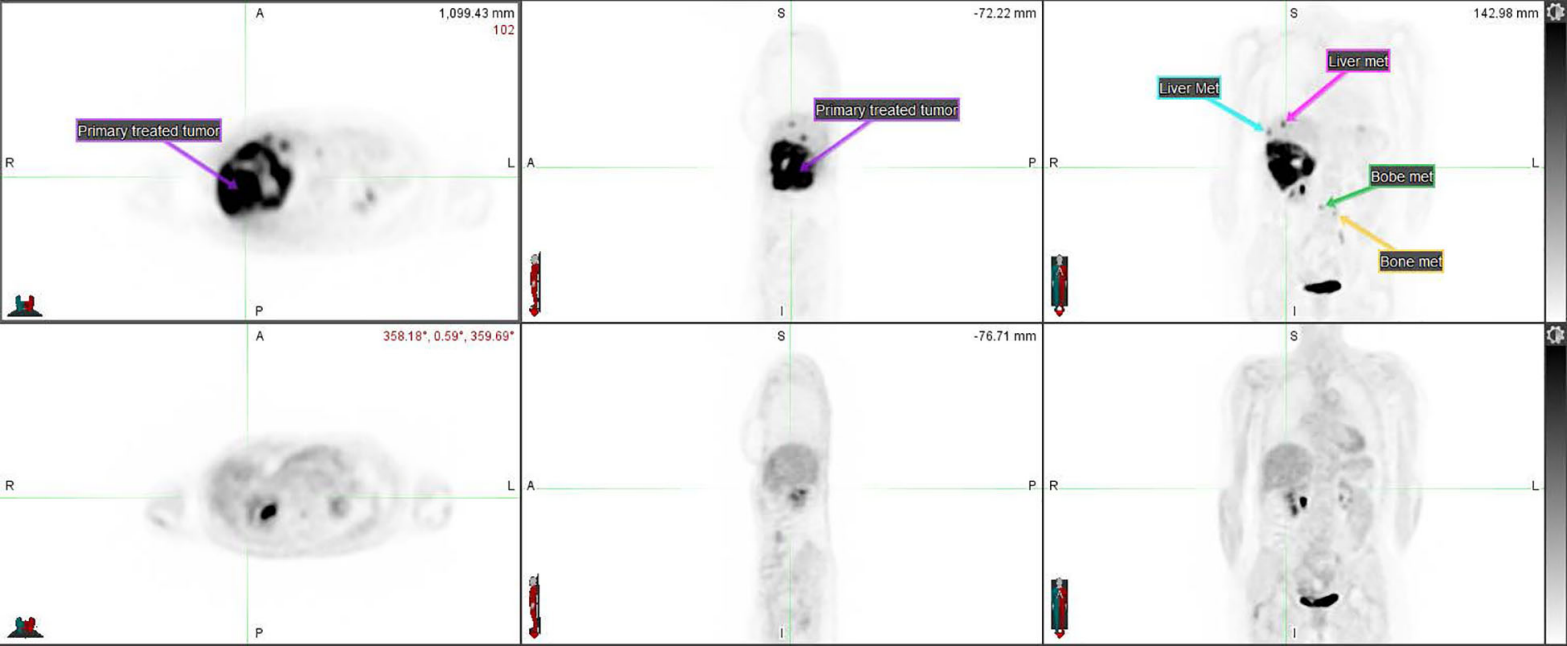
PET CT comparison of the disseminated disease before (top row) and after (bottom row) the SBRT course combined with ImT (nivolumab/relatlimab). The primary tumor treated with SBRT (compare to **Figure 2**) as well as some of the metastases are annotated on the pre-treatment imaging.

After the second cycle of *Opdualag*, the patient developed severe keratoconjunctivitis with corneal ulcerations. The patient had a history of ImT-induced hepatitis, and systemic therapy was discontinued out of concern for another severe ImT-related adverse event (irAE). Interval PET/CT 3 months after SBRT showed a CMR with no suspicious hypermetabolic activity within the surgical cavity and complete resolution of all FDG-avid lesions in the liver (including unirradiated ones) and axial spine where hypermetabolism had previously been identified (**Figure 3**).

CT chest, abdomen, and pelvis 3 months after SBRT demonstrated a decrease in the size of all liver lesions, with the segment 5 lesion measuring 2 cm in maximum diameter down from 10 cm. Six months after SBRT, the patient’s treatment response persisted despite discontinuation of all therapy for 4 months.

## Discussion

To our knowledge, this is the first description of CMR of metastatic melanoma following liver SBRT combined with relatlimab/nivolumab, notable in the setting of both visceral and osseous lesions. Furthermore, this is the first report to describe AR of SNMM, a rare (incidence rate 0.05 per 100,000) and aggressive histology.

Currently, there are no therapeutic standards specific to management of SNMM. As in most malignant melanoma, surgical resection and adjuvant radiotherapy ± systemic therapy constitute definitive treatment (3). Surgical excision is the mainstay of care, with positive or close surgical margins as risk factors for poor outcome (2, 30). Clear resection margins are nearly impossible to obtain due to the morbidity associated with aggressive sinonasal surgery. Adjuvant RT is commonly added in efforts to improve local control; however, evidence of benefit is mixed. A meta-analysis of 1,392 SNMM patients reported improved OS for patients treated with adjuvant RT (31), while a National Cancer Database (NCDB) series of 1,874 SNMMs showed no significant difference in OS. The latter series did find an association between ImT and improved OS but only in the metastatic setting (2).

The current case report describes an approach to treating SNMM with liver metastases, using SBRT to target intrahepatic disease concurrent with dual ImT, harnessing the synergism of these treatment modalities to potentiate systemic response. This combination holds the potential to impact disease control, OS, and even cure.

Cases of AR in patients with melanoma have been reported since 1975 (28). This phenomenon presents most often in patients treated with RT while undergoing immune checkpoint blockade (32). The host immune system is clearly the mediator of such non-targeted effects; it is well known that canonical immune responses to viral infection can be exploited to elicit anti-tumor CD8 T-cell responses (33), as seen in case reports describing an abscopal effect with RT after infectious diseases (like COVID-19) (34) and standard vaccination (like pneumococcal polysaccharide vaccine) (35), or in the setting of autoimmune disease (36). All of these scenarios attest to the synergistic effect that RT triggers in the immune system by releasing a large number of tumor-related antigens, which, in turn, enhance anti-tumor response mediated by CD8+ T cells (37). In addition, RT can promote antigen presentation by upregulating tumor MHC I expression and stimulating activation and maturation of dendritic cells (38). These mechanisms provide the rationale for combining local radiation with checkpoint inhibition in an effort to potentiate systemic response. There are multiple clinical and preclinical studies evaluating combinations of RT and ImT; however, there is no consensus on the ideal sequence (39) or optimal radiation dose regimen for eliciting non-targeted effects (40–42). Some preclinical investigations support radiation doses between 8 and 10 Gy per fraction (41) to achieve synergy with ImT, while others assert that the probability of stimulating AR approaches 50% with ultrahigh-dose single fraction RT, when a biologically effective dose (BED) of at least 60 Gy is delivered to the tumor using a standard alpha/beta ratio estimate of 10 (42).

The patient described in this case report developed a severe irAE after receiving two cycles of relatlimab/nivolumab, suggesting that profound tumor response and autoimmune toxicity may be linked. There is a significant tension between the desire to avoid immunosuppressive drugs in order to potentiate anti-tumor effects and the risks associated with progression of autoimmune toxicity. This provokes the challenging clinical question of whether and when to initiate immunosuppression to treat autoimmune toxicity in the setting of AR.

## Conclusion

While abscopal effects have been reported in immunogenic or “hot tumors” like melanoma, this case is the first of its kind to describe CMR in metastatic SNMM to the liver and axial spine. It is also the first to demonstrate this effect with the addition of an anti-LAG 3 immune checkpoint inhibitor. This report suggests that the combination of SBRT with ImT stimulates the adaptive immune response and is a viable path for immune-mediated tumor rejection. The mechanisms behind this response are hypothesis-generating and remain an area of active research with exceedingly promising potential.

## Data availability statement

The original contributions presented in the study are included in the article/supplementary material. Further inquiries can be directed to the corresponding author.

## Ethics statement

The studies involving human participants were reviewed and approved by University of Miami IRB. The patients/participants provided their written informed consent to participate in this study.

## Author contributions

DC prepared the manuscript. IM participated in the design of the study and the subsequent analyses and manuscript editing. BS conceptualized the idea and edited the manuscript. All authors contributed to the article and approved the submitted version.
